# Naturalization of an alien ancient fruit tree at a fine scale: Community structure and population dynamics of *Cydonia oblonga* in China

**DOI:** 10.1002/ece3.9703

**Published:** 2023-01-06

**Authors:** Yong Xie, Jiaxiang Li, Lijuan Zhao, Wenqian Liu, Qunlong Gong, Mengda Deng, Mohan Zhao, Song Huang

**Affiliations:** ^1^ College of Forestry Central South University of Forestry & Technology Changsha China; ^2^ College of Life Science and Technology Central South University of Forestry & Technology Changsha China; ^3^ Forestry Administration of Xiangtan Municipality Xiangtan China

**Keywords:** alien plant, Darwin's naturalization hypothesis, interspecific competition, naturalized plants, plagioclimax community, religious exchange

## Abstract

Naturalized plants play pivotal roles in local plant biodiversity and ecological functions; however, the drivers of naturalization remain poorly understood at a fine scale. Thus, understanding the processes of the development and dominance of alien plants in local natural habitats is of paramount importance. In the present study, we report for the first time the naturalization of *Cydonia oblonga* in China based on community structure and population dynamics at a fine scale. We conducted a comprehensive survey of the species through field community investigations, interviews, and a literature review. *Cydonia oblonga* is an ancient fruit tree with a long introduction history of over 4500 years worldwide and a cultivation history of over 2500 years in China. We analyzed *C. oblonga* community structure using the spatiotemporal substitution method and quantitatively analyzed population dynamics using a static life table, survivorship curve, and time series model to explore the naturalization processes. The following results were obtained. (i) The community comprised 31 coexisting vascular plant species (16 woody and 15 herbaceous species) belonging to 28 genera in 20 families. Rosaceae and Asteraceae were the two most dominant families. (ii) All individuals in the shrub layer as well as the *C. oblonga* population exhibited a roughly inverted J‐shaped basal diameter distribution. A complete age structure was noted, and the survival curve was classified as Deevey type II. According to time series analysis, the population is estimated to increase in the future, specifically of medium and large individuals. (iii) Religious exchange, potent resource competitiveness, and similarity with the native habitat may be the major drivers of the introduction and successful naturalization of *C. oblonga*. These results suggest that alien species closely related to native ones are more likely to invade, naturalize, and dominate communities in local habitats.

## Graphical abstract

We report for the first time the naturalization of *Cydonia oblonga* in China and East Asia based on community structure and population dynamics at a fine scale. We conducted a comprehensive survey of the species through field community investigation, interviews, and literature information. The results suggest that alien species closely related to native species are more likely to invade, naturalize, and become dominant species in local habitats, which strongly supports the “pre‐adaptation hypothesis” at a fine scale.

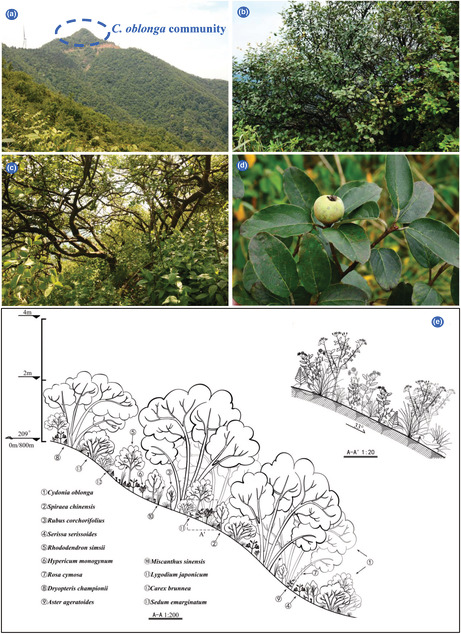

## 1 INTRODUCTION

With the development of human society and the convenience of international exchange of goods, the introduction and spread of alien plants have been unstoppable worldwide. The intentional introduction is the major mode of transmission of alien plants, particularly of species with high economic value (e.g., fruit trees) that are favored by humans (Qian & Sandel, 2021; van Kleunen et al., 2020; Xu et al., 2019). Alien plants that consistently reproduce and sustain populations in natural habitats for a relatively long time (generally over 10 years) without direct intervention by humans are regarded as naturalized species (Jiang et al., 2011; Pyšek et al., 2004; Richardson et al., 2000), and such species may spread out of control to turn invasive, producing potentially negative effects on local biodiversity and ecological functions (Pyšek et al., 2020). Therefore, in recent decades, an increasing number of ecologists have focused on the identification of naturalized plants and the potential drivers of their naturalization (Essl et al., 2019; Pyšek et al., 2020). Thanks to these efforts, checklists of naturalized plants are available for many countries and regions. For instance, van Kleunen et al. (2015) established a global naturalized plant database, which includes over 13,000 species, accounting for 4% of the known vascular plants worldwide (Pyšek et al., 2017). Furthermore, approximately 1099, 787, 525, and 291 naturalized plant species have been recorded in China (Xu et al., 2019), India (Khuroo et al., 2012), Brazil (Zenni, 2015), and Ghana (Ansong et al., 2019), respectively.

The success of naturalized plants is closely linked to the similarities and differences in niches and the genetic relationships among alien and native species (Li, Cadotte, et al., 2015). Two seemingly contradictory hypotheses regarding the phylogenetic relatedness of native and alien species affecting the naturalization process have been proposed by Charles Darwin (Darwin, 1859; Park et al., 2020; Qian & Sandel, 2021). According to Darwin's “naturalization hypothesis,” alien species that are phylogenetically distinct from the native flora are more likely to naturalize because of their ability to exploit the unoccupied ecological niches in native communities. Meanwhile, according to Darwin's “pre‐adaptation hypothesis,” exotic species that are closely related to indigenous ones may establish more successfully because they share an affinity to the local environment (Cadotte et al., 2018; Park et al., 2020; Qian & Sandel, 2021). Therefore, according to both views, the environmental adaptability of alien species and their interactions with native species are the key drivers of the successful naturalization of these plants (Li, Cadotte, et al., 2015). Given the differences in research objects, spatial scales, and invasion stages, similar proportions of previous studies based on regional (Daehler, 2001; Rejmánek, 1996) or continental (Diez et al., 2009; Mack, 1996) observations have supported the above contradictory hypotheses (Li, Cadotte, et al., 2015). However, in a meta‐analysis of existing literature on plants at the regional and local scales, Ma et al. (2016) demonstrated that the invader–native relatedness closely depends on the spatial scale and invasion stage. Nonetheless, species frequently interact and compete with one another at finer scales, at which they share the same environment and are subjected to the same biotic interactions (Li, Cadotte, et al., 2015; Tilman, 2004). Overall, the population dynamics and roles of alien species in local natural communities determine their success in the ecosystem (Ma et al., 2016), and these parameters can therefore be applied to judge whether a given alien species is naturalized (Jiang et al., 2011; Pyšek et al., 2004; Richardson et al., 2000). In this context, the community structure and population dynamics of species must be explored at fine scales to study the naturalization process and its drivers in exotic plants.

**FIGURE 1:**
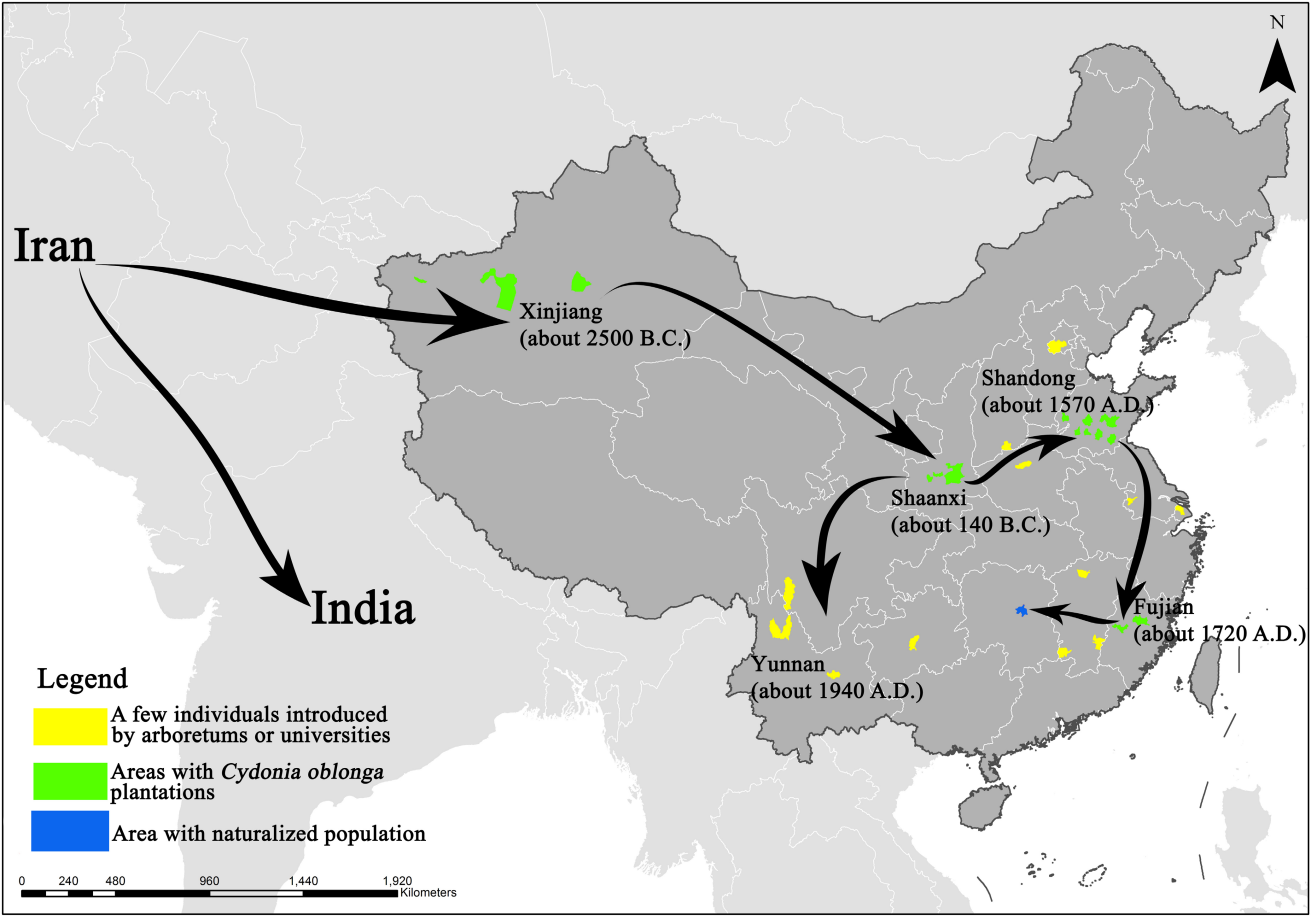
Introduction history, route, and cultivation distribution of *Cydonia oblonga* in China. Black arrows show the introduction routes. The numbers in brackets indicate the historical time of introduction in different regions. Colored patches indicate the counties where *C. oblonga* has been introduced in China. Yellow, green, and blue patches represent the counties with a few individuals introduced by arboretums and universities, *C. oblonga* plantations, and naturalized populations, respectively.

*Cydonia oblonga* Mill. (commonly called quince) is an ancient fruit tree of the Rosaceae family, with high value in food, medicine, spices, industry, and horticulture (Luo et al., 2018). It originated in the region between Dagestan and Talysh and has spread to several countries, creating multiple diversity centers along its distribution route (Abdollahi, 2019). The domestication and cultivation history of *C. oblonga* can be traced back over 4000 years (Abdollahi, 2019). Approximately 2500 years ago, the species was first introduced from Persia to the Xinjiang Province of China; subsequently, it spread to eastern China along the Yellow River and finally to the south of the Yangtze River over 300 years ago (Figure 1; Liu & Liu, 1982; Liu & Wang, 2016). Most previous studies have focused on the horticultural and medicinal properties of quince, and only a few reports are available on its self‐regeneration and maintenance mechanisms in natural habitats.

In July 2021, during our expedition to the Baozhong Mountain in Xiangxiang County, a community dominated by *C. oblonga* was identified on an inaccessible rocky hilltop, representing the first record of this species in Hunan Province, China. The concerned habitat is unfavorable for cultivation, and the community formed by individuals of uneven ages in this habitat markedly differs from the conventional or abandoned semi‐natural plantations formed by individuals of even age (Luo et al., 2018; Zhang, 2006). These unusual observations prompted us to trace the introduction and development of this alien plant. To this end, based on field investigation of the identified community combined interviews and historical literature review, we aimed (i) to investigate the species composition, structure, and developmental trends of a community dominated by *C. oblonga* in the local natural habitat; (ii) to estimate whether the studied population is naturalized and acts a component of the local ecosystem; and (iii) to discuss the potential drivers of the introduction and establishment processes of these alien ancient fruit trees in China.

## 2 MATERIALS AND METHODS

### 2.1 Study site

The present study was conducted in the Baozhong Mountain (27°48′–27°52′ N, 112°12′–112°13′ E), Xiangxiang County, Hunan Province, China. Baozhong is an isolated mountain, with an altitude ranging from 185 to 802 m above sea level. The climate is mid‐subtropical humid, with the mean annual ambient temperature of 15°C, mean annual precipitation of 1240–1360 mm, and annual frost‐free period of 262–275 days. The soil types include red, yellow‐red, and yellow soil, with an average thickness of 40–80 cm, developed on the slate, shale, and sandstone rock. The zonal vegetation is a mid‐subtropical evergreen broad‐leaved forest. However, most of the original vegetation was severely damaged before the 1980s and has been replaced by *Cunninghamia lanceolata* plantations, *Phyllostachys edulis* forests, secondary broad‐leaved forests, and shrublands. The identified *C. oblonga* community occupies a rocky hilltop of the mountain, ranging in altitude from 750 to 802 m. The long religious history of this region is reflected by the popular Baoen Temple built in A.D. 190 (Editorial Committee of Xiangxiang County Chronicles, 1993); the trees and forests around the temple are deified and protected because of people's religious beliefs. At present, a small area of the old‐age forest (approximately 2 ha) remains, harboring over 80 woody species with the largest diameter at breast height of over 100 cm.

### 2.2 Field investigation

During our first visit to the study site in July 2021, we collected specimens with fruits and recorded the general habitat information. Based on the morphological traits of the specimens and consultation with an expert on the Chinese subfamily Maloideae of Rosaceae, we identified the species as *C. oblonga* Mill. The species is characterized by leaf blades bearing densely villous abaxial surfaces with conspicuous veins, five‐loculed inferior ovaries, and many‐seeded pomes, developed solitary at the apices of leafy branchlets, densely tomentose, with persistent reflexed sepals (Gu & Stephen, 2003). The voucher specimens (collection number: Li Jiaxiang and Xie Yong BML013) are deposited in the Forest Herbarium of Central South University of Forestry and Technology (Herbarium code: CSFI).

Following the accurate identification of *C. oblonga*, we undertook a detailed community survey using 20 quadrats measuring 5 m × 5 m in August 2021. This quadrat size has been previously applied in surveys of shrub communities (Fartyal et al., 2022; Malakar & Joshi, 2020; Wang et al., 2020). Within each quadrat, clumped shrubs with an average height of 1.73 m (maximum height = 3.9 m) were the dominant species in the community. Woody plants with branches more than 5 cm above the ground were regarded as independent individuals, and their taxonomic name, basal diameter (BD, trunk diameter at 5 cm above the ground), height (H), and crown width were recorded (Li, Zhang, et al., 2015). Additionally, the taxonomic name, abundance, coverage, and average height of herbaceous species were recorded within three quadrats measuring 1 m × 1 m, arranged diagonally at equal intervals within each 5 m × 5 m quadrat. Furthermore, the names of species outside the sampling plots were recorded.

Furthermore, we investigated the religious background and introduction history of *C. oblonga* to better understand its development in the study region by browsing the chronicles of Xiangxiang County (Editorial Committee of Xiangxiang County Chronicles, 1993) and consulting with the elderly who were aware of the history of the local temples. The questionnaire included queries on the temple name, its time of establishment and destruction, its abbot, its monk's preference for flowers and trees at the time, the introduction of plants in temples, and Buddhist cultural exchanges across temples and other provinces. Finally, eight effective questionnaires were collected by consulting with the local elders who were 80 years of age or older.

### 2.3 Data analysis

To determine species assembly, species dominance, and *C. oblonga* community dynamics and stability, we divided the studied community into two synusia of shrub and herb layers according to species life forms. Density, mean BD, mean H, coverage (C), and basal area (BA) were calculated for each species within the shrub layer, and abundance, mean H, frequency, and coverage (C) were calculated for each species within the herb layer. The relative importance value (RIV) for each species was measured as relative density, relative BA, and relative coverage in the shrub layer as well as relative coverage, relative abundance, and relative frequency in the herb layer, standardized on a percent basis. To generate size‐frequency and height‐frequency distributions, all individuals within the shrub layer were grouped into 2 cm size classes of BD and 0.5 m height classes of H (Li, Zhang, et al., 2015).

To determine the population dynamics, age structure analysis, static life table, survivorship curve, and time series analysis were applied (Li et al., 2020; Tuo et al., 2021). Age structure reflects the demographic status, dynamic trends, dominance, and roles of the population in the community (Díaz et al., 2000). The static life table and survival curve can directly present the proportion of surviving individuals, deaths, survivorship trends, and other important demographic characteristics and are crucial means to evaluate the population dynamics of long‐lived, slow‐growing woody plants (Díaz et al., 2000; Harcombe, 1987; Li et al., 2020). Time series analysis was used to forecast the age structure and population trends in the future (Li et al., 2020).

First, we used the spatiotemporal substitution method to divide the age classes according to BD, which reflect the age structure and survival status of dominant species in shrublands (García et al., 1999; McCarthy & Weetman, 2006). Based on BD, we divided the individuals into eight age classes and four growth stages. Detailed classification criteria are presented in Table 1. Second, to reflect the changes in natality and mortality rates of the population, we compiled the static life table of *C. oblonga* based on the specific age distribution frequency over multiple generations overlapping in the population; the number of surviving individuals fluctuated very highly across the different age classes (Wu et al., 2010). The static life table was generated as follows (Deevey Jr., 1947; Farahat, 2020; Pielou, 1977):

**TABLE 1:** The classification of growth stages and age classes

Growth stages	Age classes	BD classes	BD (cm)
Seedlings	A_1_	I	(0, 2)
Small trees	A_2_	II	[2, 4)
	A_3_	III	[4, 6)
Medium trees	A_4_	IV	[6, 8)
	A_5_	V	[8, 10)
Large trees	A_6_	VI	[10, 12)
	A_7_	VII	[12, 14)
	A_8_	VII	[14, 16)

*Note*: BD is the basal diameter.

First, the standardized number of survivors (*l*_*x*_) in age class *A*_*x*_ was calculated as follows:

(1) $$l_{x}=\left(A_{x}/A_{0}\right)\times 1000$$

Next, the proportion of original cohort dying during each stage (*d*_*x*_) was calculated as follows:

(2) $$d_{x}=l_{x}-l_{x+1}$$

Then, the stage‐specific mortality rate (*q*_*x*_) was calculated as follows:

(3) $$q_{x}=\left(A_{x}/l_{x}\right)\times 100\%$$

The average proportion of surviving individuals at the age *x* (*L*_*x*_) was calculated as follows:

(4) $$L_{x}=\left(l_{x}+l_{x+1}\right)/2$$

The total number of surviving individuals in age class *x* and beyond (*T*_*x*_) was calculated as follows:

(5) $$T_{x}=L_{x}+L_{x+1}+L_{x+2}+\ldots +L_{x+n}$$

The probability of an individual surviving for “*x*” number of years beyond a given age *x* (*e*_*x*_), which reflects the average survival ability of individuals in age class *x*, was calculated as follows:

(6) $$e_{x}=T_{x}/l_{x}$$

The rate or degree of mortality in any cohort, which reflects the killing power (*K*_*x*_), was calculated as follows:

(7) $$K_{x}=\ln\;l_{x}-\ln\;l_{x+1}$$

The survival rate (*S*_*x*_) was calculated as follows:

(8) $$S_{x}=l_{x+1}/l_{x}$$

Third, to reflect the survival status of individuals in each age class, with age class (*x*) on the *X*‐axis and logarithmic standardized survival number (ln*l*_*x*_) on the *Y*‐axis, we created a survivorship curve based on the exponential (*N*_x_ = *N*_o_ *e* ^−bx^) and power (*N*_x_ = *N*_o_ *x* ^−b^) functions proposed by Hett and Loucks (1976). According to the coefficient of determination and *F*‐value, the optimal model was determined. Then, based on model fitting, the type of the curve was determined to reflect the survival status of the population (Chen et al., 2021). The survivorship curve can be divided into three types, namely upward concavity (Deevey type I), straight line (Deevey type II), and downward concavity (Deevey type III) curves, which respectively indicate that mortality at the seedling stage is lower than that at the mature stage, mortality at each stage is equal, and mortality at the seedling stage is higher than that at the mature stage (Deevey Jr., 1947).

Fourth, we used the survival rate function *S*_(*i*)_, cumulative mortality rate function *F*_(*i*)_, mortality density rate function *f*_(*i*)_, and hazard rate function *λ*_(*i*)_ to analyze the population dynamics (Wu et al., 2010). In the following equations, *S*_*i*_ is the survival rate and *h*_*i*_ is the age‐class interval.

(9) $$S_{\left(i\right)}=S_{1}\times S_{2}\times S_{3}\times \ldots \times S_{i}$$

(10) $$F_{\left(i\right)}=1-S_{i}$$

(11) $$f_{\left(i\right)}=\left(S_{i-1}-S_{i}\right)/h_{i}$$

(12) $$\lambda_{\left(i\right)}=2\left(1-S_{i}\right)/\left[h_{i}\left(1+S_{i}\right)\right]$$

Finally, a time series model was used to predict the population developmental trends in the next two, four, six, and eight age classes. In Equation 13, *n* is the prediction time, *t* is the age class, *M*_*t*_ is the population size in the *n*th year, and *X*_*k*_ is the population size in the *k*th age class.

(13) $$M_{t}=\frac{1}{n}\times \sum_{k=t-n+1}^{t}X_{k}$$

Furthermore, to analyze the phylogenetic relationships between *C. oblonga* and other woody species in the community, we generated a cladogram of 17 species in the shrub layer using V.PhyloMaker, a freely available R package designed to generate the phylogenies of vascular plants (Jin & Qian, 2019).

## 3 RESULTS

### 3.1 Community composition and structure

A total of 31 species of vascular plants belonging to 28 genera in 20 families were recorded in the *C. oblonga* community, including 3 families, 3 genera, and 3 species of ferns as well as 17 families, 25 genera, and 28 species of angiosperms (Tables 2 and 3). The most dominant families were Rosaceae (with three genera and six species) and Asteraceae (with four genera and four species). In addition, 16 woody plants (51.61% of all plants), including 13 deciduous (81.25% of all woody plants) and only 3 (1.75% of all woody plants) evergreen woody plants, were recorded.

**TABLE 2:** Density, mean basal diameter (BD), mean height (H), basal area (BA), and the relative importance value (RIV) of each species in the shrub layer of *Cydonia oblonga* community

Species	Density (stems ha^−1^)	Mean BD (cm)	Mean H (m)	BA (m^2^ ha^−1^)	RIV (%)
*Cydonia oblonga*	3850	4.88	1.73	10.93	67.12
*Spiraea chinensis*	2650	0.77	0.92	0.13	9.97
*Rhododendron simsii*	1150	1.12	0.99	0.12	4.97
*Serissa serissoides*	1550	0.47	0.35	0.03	4.10
*Rubus corchorifolius*	1100	0.60	0.93	0.04	3.30
*Hypericum monogynum*	950	0.43	0.40	0.01	2.49
*Lespedeza bicolor*	800	0.38	0.30	0.01	2.13
*Rubus parvifolius*	400	0.44	0.90	0.01	1.24
*Rosa cymosa*	350	0.79	1.06	0.02	1.19
*Ilex chinensis*	300	0.58	0.75	0.01	0.97
*Mallotus apelta*	50	3.20	1.50	0.04	0.70
*Castanea seguinii*	150	0.80	1.00	0.01	0.55
*Ligustrum leucanthum*	150	0.43	0.47	<0.01	0.47
*Lonicera japonica*	100	0.50	0.30	<0.01	0.26
*Smilax china*	100	0.30	0.60	<0.01	0.25
*Viburnum setigerum*	50	0.30	0.30	<0.01	0.15
*Rubus tephrodes*	50	0.40	0.30	<0.01	0.14
Total	13,750	1.84	1.00	11.36	100.00

**TABLE 3:** Mean height (H), mean coverage (C), relative abundance (RA), relative coverage (RC), and relative importance value (RIV) of each species in the herb layer of *Cydonia oblonga* community

Species	Mean H (m)	Mean C (%)	RA (%)	RC (%)	RIV (%)
*Carex brunnea*	0.21	18.92	50.55	62.62	49.49
*Dryopteris championii*	0.25	2.75	12.09	9.10	11.97
*Aster ageratoides*	0.41	2.21	13.19	7.31	11.73
*Miscanthus sinensis*	0.73	4.17	6.59	13.79	9.74
*Lygodium japonicum*	0.13	0.33	4.40	1.10	3.79
*Sedum emarginatum*	0.05	0.08	4.40	0.28	3.52
*Woodwardia japonica*	0.20	0.75	2.20	2.48	2.54
*Liriope spicata*	0.30	0.42	2.20	1.38	2.17
*Viola inconspicua*	0.05	0.08	2.20	0.28	1.80
*Senecio scandens*	0.15	0.25	1.10	0.83	1.62
*Conyza japonica*	0.25	0.25	1.10	0.83	1.62
Total	0.26	30.21	100	100	100

**FIGURE 2:**
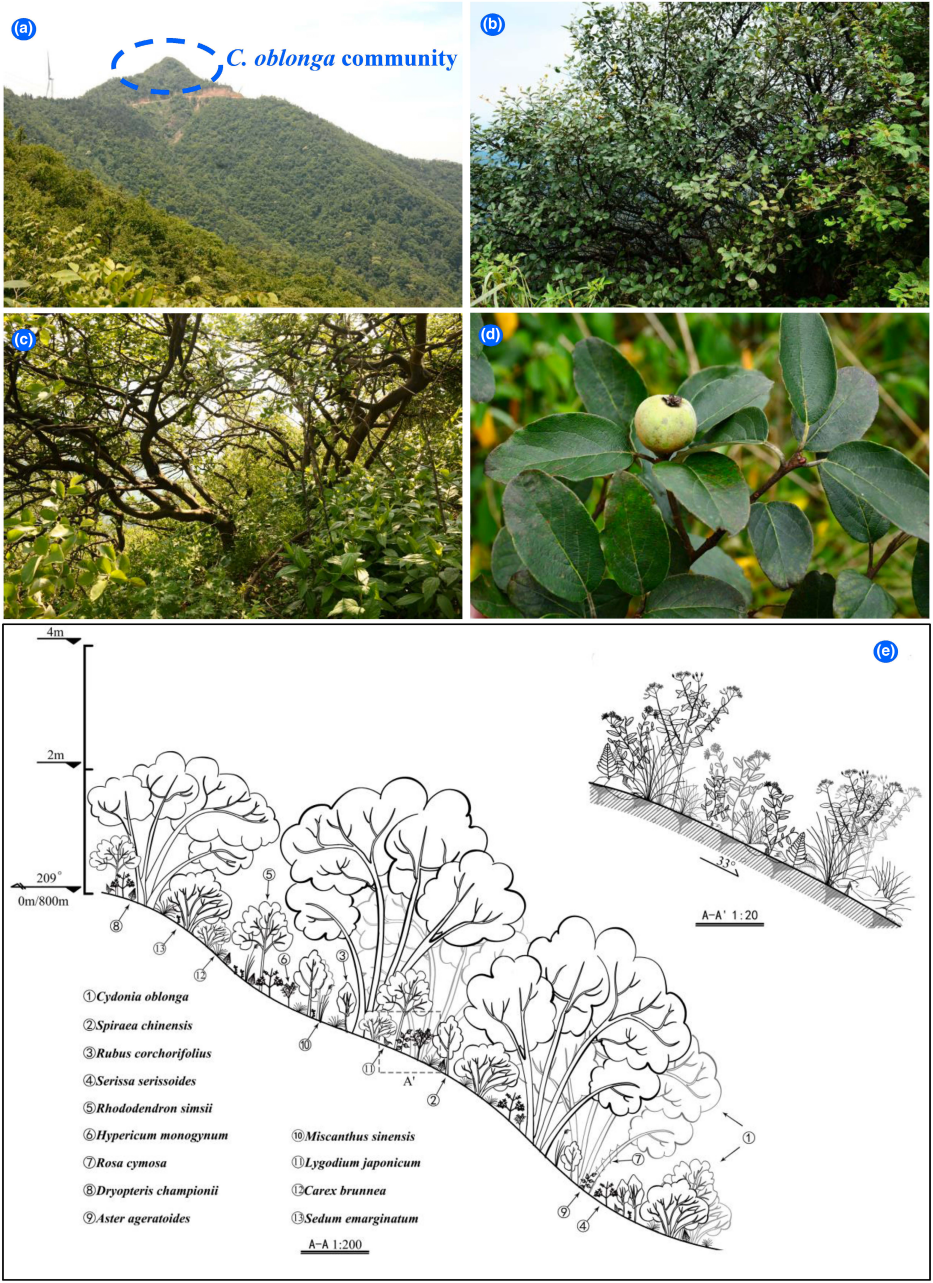
Illustration of habitat and community structure of *Cydonia oblonga* community in the Baozhong Mountain, Hunan province, China, at an altitude of 800 m above the sea level. (a) *C. oblonga* community at the top of the mountain; (b) Community physiognomy; (c) Vertical community structure; (d) Fruit branches; (e) Scientific illustration of vertical structure of the *C. oblonga* community which illustrates species composition, community height, interspecific relationships, and topography.

According to the height of synusia, the community was clearly divided into shrub and herb layers, and the shrub layer was subdivided into quince and dwarf shrub layers (Figure 2). The quince layer was dominated by *C. oblonga*, with an RIV of 67.12%, a coverage of >60.00%, and an average height of 1.73 m. In contrast, the coverage of dwarf shrub layer was approximately 45.00%; its average height was 0.73 m; and it was dominated by *Spiraea chinensis* (10.51%), *Rhododendron simsii* (5.90%), *Serissa serissoides* (4.55%), *Rubus corchorifolius* (3.62%), and other species (Table 2). The coverage of herb layer was approximately 30%, and its average height was 0.26 m. *Carex brunnea* was the dominant species in the herb layer, with the maximum RIV of 49.49%, followed by *Dryopteris championii* (11.97%), *Aster trinervius ageratoides* (11.73%), and other species (Table 3).

**FIGURE 3:**
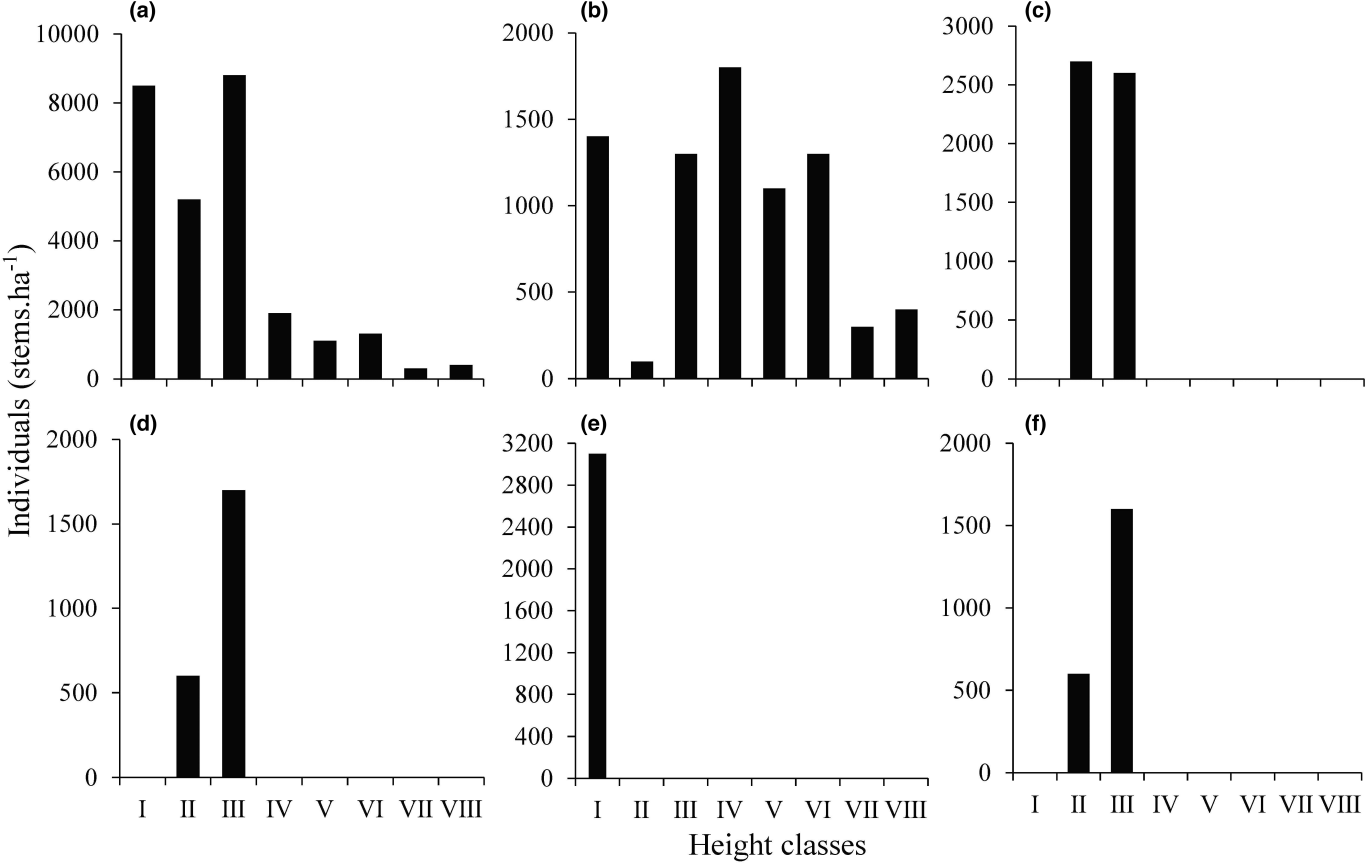
The height classes of all individuals and the top five species with important values in the shrub layer of *Cydonia oblonga* community. (a) All individuals; (b) *Cydonia oblonga*; (c) *Spiraea chinensis*; (d) *Rhododendron simsii*; (e) *Serissa serissoides*; (f) *Rubus corchorifolius*. All individuals of shrub species are grouped into 0.50 m size classes of height which expresses as class H_1_, H_2_, H_3_, H_4_, H_5_, H_6_, H_7_, and H_8_.

The height of most individuals in the shrub layers was below 1.50 m (81.82% of the total individuals), and their number decreased sharply with increase in individual height. Approximately 32% individuals were between 1 and 1.50 m tall (Figure 3a). The height of most quince individuals (30.51%) was 2–2.50 m, with the highest individual being 3.80 m tall (Figure 3b). Meanwhile, 23.72% individuals were regenerating seedlings, with an average height of 0.20 m. The other dominant species were relatively shorter and were concentrated in only one or two height classes below 1.50 m (Figure 3c–f).

**FIGURE 4:**
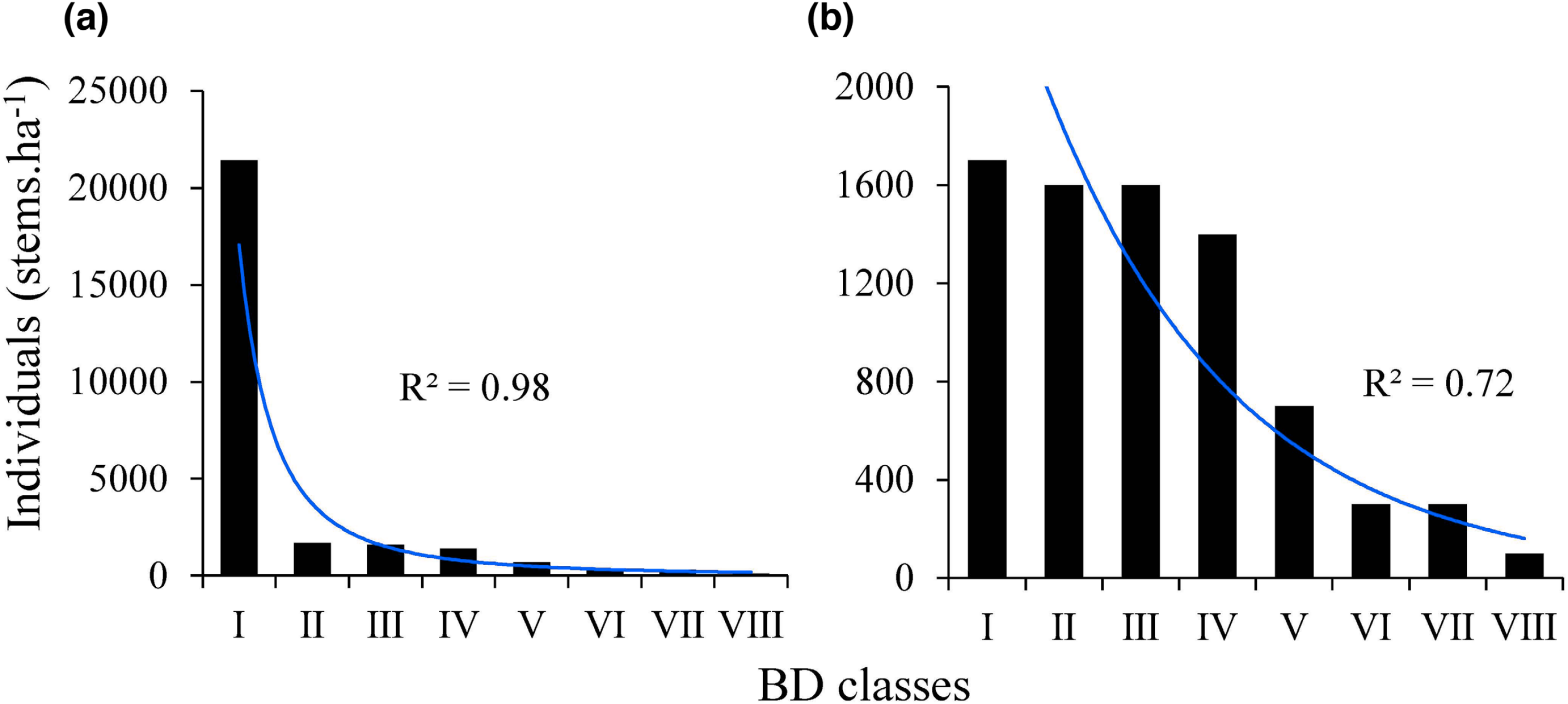
The basal diameter (BD) structure of all shrub species (a) and *Cydonia oblonga* population (b). All individuals of shrub species are grouped into 2 cm size classes of BD which are expressed as class I, II, III, IV, V, VI, VII, and VII. Blue lines indicate population change trends with *p* < .001 of regression significance level.

A roughly inverted J‐shaped distribution of BD was noted among individuals of the shrub layer, with dwarf shrubs and *C. oblonga* seedlings (BD < 2 cm) accounting for 10,700 stems ha^−1^, that is, 77.82% of the total number of individuals in the shrub layer (Figure 4a). *Cydonia oblonga* dominated the other BD classes and showed a roughly inverted J‐shape distribution of BD (Figure 4b).

### 3.2 Population dynamics of *Cydonia oblonga*

**FIGURE 5:**
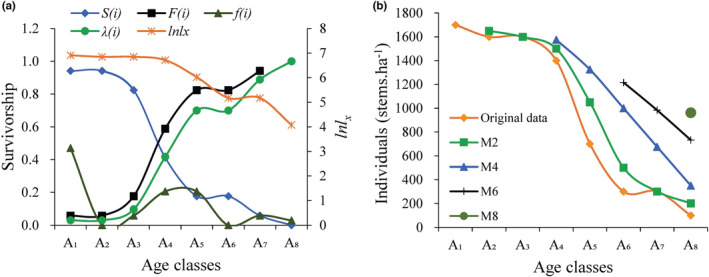
The survival rate, cumulative mortality rate, mortality density rate and hazard rate function curves (a), and the time sequence prediction (b) of *Cydonia oblonga* population. *X* represents the age classes which are divided into class A_1_, A_2_, A_3_, A_4_, A_5_, A_6_, A_7_, and A_8_ according to the basal diameter class; *A*_*x*_, survival number; *l*_*x*_, standardized survival number; *d*_*x*_, death number; *q*_*x*_, standardized mortality rate; *L*_*x*_, span life; *T*_*x*_, total life; *e*_*x*_, life expectancy; *K*_*x*_, the disappearance rate; *S*_*x*_, the survival rate. Using the time sequence model, the population developmental trend in the next A_2_ (M2), A_4_ (M4), A_6_ (M6), and A_8_ (M8) age classes were predicted.

With increasing age, the standardized survival number (*l*_*x*_) of the *C. oblonga* population decreased gradually; similarly, life expectancy (*e*_*x*_) showed a decreasing trend, albeit fluctuating slightly within age class A_6_ (Table 4). The standardized mortality rate (*q*_*x*_) and disappearance rate (*K*_*x*_) were zero in age classes A_2_ and A_6_, respectively. Excluding age classes A_2_ and A_6_, *q*_*x*_ and *K*_*x*_ showed an increasing trend, which was closely related to *e*_*x*_. The optimal model was an exponential function (*y* = 2408.2 e^−0.404x^, *R* ^2^ = .8738), indicating that the survivorship curve belonged to Deevey type II (Figure 5a).

**TABLE 4:** Static life table of *Cydonia oblonga* population

Age classes	BD classes	*A*_x_	*l*_x_	*lnl*_x_	*d*_x_	*q*_x_	*L*_x_	*T*_x_	*e*_x_	*K*_x_	*S*_x_
A_1_	I	1700	1000	6.91	58.82	0.06	970.59	4029.41	4.03	0.06	0.94
A_2_	II	1600	941.18	6.85	0	0.00	941.18	3058.82	3.25	0	1
A_3_	III	1600	941.18	6.85	117.65	0.13	882.35	2117.65	2.25	0.13	0.88
A_4_	IV	1400	823.53	6.71	411.76	0.50	617.65	1235.29	1.50	0.69	0.50
A_5_	V	700	411.76	6.02	235.29	0.57	294.12	617.65	1.50	0.85	0.43
A_6_	VI	300	176.47	5.17	0	0.00	176.47	323.53	1.83	0	1
A_7_	VII	300	176.47	5.17	117.65	0.67	117.65	147.06	0.83	1.10	0.33
A_8_	VII	100	58.82	4.07	—	—	29.41	29.41	0.50	—	—

Abbreviations: *A*_*x*_, survival number per hector; BD, basal diameter; *d*_*x*_, death number; *e*_*x*_, life expectancy; *K*_*x*_, the disappearance rate; *L*_*x*_, span life; *l*_*x*_, standardized survival number; *q*_*x*_, standardized mortality rate; *S*_*x*_, the survival rate; *T*_*x*_, total life; *x*, age class.

Overall, the *C. oblonga* population dynamics were characterized by stability at the early stage, decline at the intermediate stage, and physiological decline at the later stage. Survivorship curves showed that the survival rate, *S*_(*i*)_ of the *C. oblonga* population decreased, whereas the cumulative mortality rate, *F*_(*i*)_ and hazard rate, *λ*_(*i*)_ increased. The amplitudes of the decrease in *S*_(*i*)_ and increase in *F*_(*i*)_ and *λ*_(*i*)_ in age classes A_3_ *–*A_5_ were greater than those in the other classes. The mortality density rate, *f*_(*i*)_ fluctuated as a whole, with the maximum value recorded in age class A_1_, followed by A_4_ and A_7_ (Figure 5a). Moreover, time series analysis indicated that the population will increase greatly after A_4_, although the increase in A_2_–A_4_ is predicted to be lower in the future (Figure 5b).

### 3.3 Phylogenetic relationships among woody species

**FIGURE 6:**
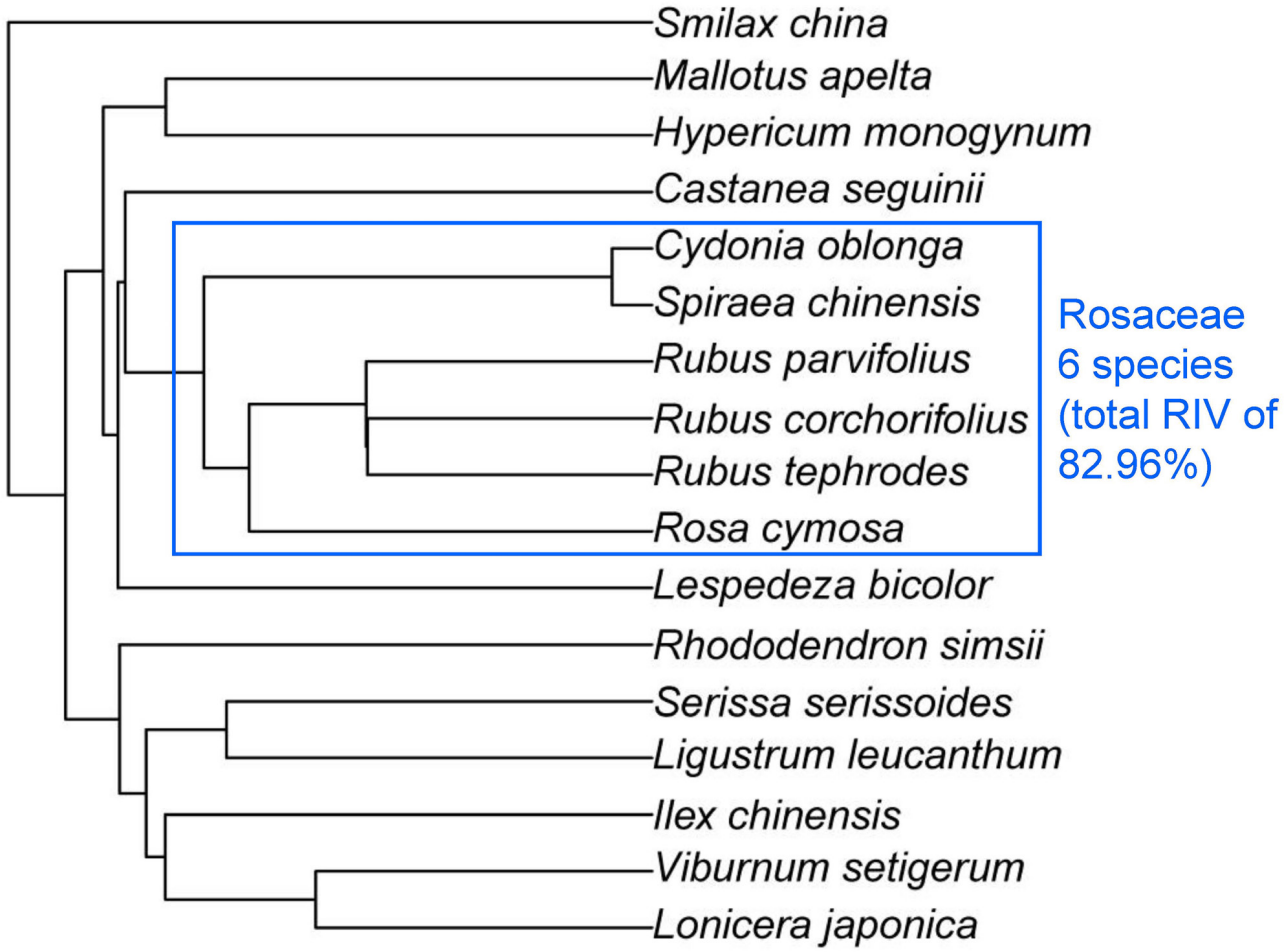
Phylogenetic relationship of 17 woody species from the *Cydonia oblonga* community in Baozhong Mountain, Hunan, China. The blue square indicates all 6 species belonging to Rosaceae with 82.96% of total relative important value (RIV) in the shrub layer.

Phylogenetic analysis showed that *C. oblonga* is closely related to five other native species (*Spiraea chinensis*, *Rosa cymosa*, *Rubus corchorifolius*, *Rubus parvifolius*, and *Rubus tephrodes*) of the Rosaceae family, specifically to *Spiraea chinensis* (Figure 6). All six Rosaceae species shared 82.96% RIV, while *C. oblonga* and *S. chinensis* shared 77.09% RIV among all shrub species (Figure 6, Table 2).

## 4 DISCUSSION

### 4.1 Community structure and population dynamics

The key criterion for identifying an alien species as naturalized is whether it can reproduce in the wild and form a self‐sustaining population independent of direct human intervention (Blackburn et al., 2011; Richardson et al., 2000). During our recent field investigation, we identified, for the first time, a wild *C. oblonga* population at the top of the Baozhong Mountain in Hunan, China. Due to steep terrain and rocky habitat, human intervention, such as agricultural activities and artificial afforestation, is extremely unlikely in this region; additionally, the region has experienced natural interference only in the past. From the perspective of the RIV composition (Table 3) and height class structure (Figure 3), *C. oblonga* was the most dominant population, shaping the community structure and environment. The roughly inverted J‐shape BD distribution (Figure 4) indicates the excellent reproductive and recruitment potential of the identified *C. oblonga* population (Birhanu et al., 2018; Didita et al., 2010). Moreover, from the survival curve of Deevey type II, values of *e*_*x*_ and *q*_*x*_ in the static life table (Table 4), and results of time series analysis, the seedlings present high viability and low mortality (Figure 5b). Furthermore, quantitative analysis of population dynamics indicated that the population may self‐regenerate and remain stable for a long time in the natural habitat, which is consistent with the criterion of species naturalization (Jiang et al., 2011; Pyšek et al., 2017; Richardson et al., 2000). Therefore, we speculate that *C. oblonga* has been naturalized in the Baozhong Mountain. Furthermore, according to the Global Naturalized Alien Flora (GloNAF; https://glonaf.org/; van Kleunen et al., 2019), *C. oblonga* has been naturalized in 50 regions of 15 countries in Europe, Southern America, Northern America, Africa, and Australasia. However, it has not been included in the list of naturalized species in China as yet (Xu et al., 2019; Yan et al., 2019). Accordingly, the present study is the first to infer the naturalization of *C. oblonga* in China.

Zonal vegetation in the subtropics of China constitutes subtropical evergreen broad‐leaved forests, which are shaped by climate at the regional scale, representing the succession direction of plant communities without anthropogenic interference (Bugmann & Solomon, 2000; Chen et al., 2010). Nevertheless, local processes, including habitat filtering, species interactions, and dispersal limitation, ultimately determine the species composition and structural dynamics of local communities; therefore, plagioclimax communities shaped by topography, soil, and disturbance in unique local habitats are strong and stable (Solon et al., 2007). The identified *C. oblonga* community is distributed on steep rocky slopes at the top of the mountain (Figure 2a). Here, the soil layer is shallow and solar radiation is intense. To adapt to such an adverse habitat, most species are short, deciduous, light‐demanding, and with thick, hairy leaves (Bin et al., 2012; Zhu & Wei, 2016). *Cydonia oblonga* showed longevity and has successfully established as a dominant population in the community. Meanwhile, *Carex brunnea*, with shade‐tolerant, perennial, and evergreen life‐history traits, dominates the herb layer. However, from the H and BD class distributions, other shrubs belong to dwindling populations in the community (Figures 3 and 4). Owing to the limited light availability in the dwarf shrub layer, the growth of these individuals is seriously restricted, resulting in the gradual withdrawal of their populations from the community (Barbier et al., 2008). Therefore, the identified *C. oblonga* community appears to be at the intermediate or later stage of succession and may develop into a plagioclimax community under adverse habitat conditions on the mountaintop.

### 4.2 Potential drivers of *Cydonia oblonga* naturalization

Introduction, establishment, naturalization, and subsequent impact are the four major stages of the invasion process of alien species (Li, Cadotte, et al., 2015; Richardson et al., 2000). Although plant diversity in Hunan has been cataloged for over a century, no information is available on *C. oblonga* during this period (Qi & Yu, 2002); therefore, the introduction history of this alien species is the primary problem that must be addressed. For thousands of years, *C. oblonga* was not only significant as food, medicine, and spice but also intentionally introduced into ancient royal gardens, indicating its rarity and uniqueness at the time in China. Through interviews and queries on history, we obtained some crucial information related to the introduction of *C. oblonga* (Figure 1). An influential Buddhist abbot of the Baoen Temple once advocated his disciples to bring several seedlings of local valuable trees to the temple for cultivation when they visited their native places (Editorial Committee of Xiangxiang County Chronicles, 1993). Moreover, frequent Buddhist exchanges between Hunan and Fujian have been documented, indicating over 300 years of *C. oblonga* cultivation history (Zhang, 2006). Such exchanges have since been carried forward over generations, and some elderly people around the Baozhong Mountain have the memory of such events even today. Therefore, we speculate that the identified *C. oblonga* population might have been introduced from Fujian and dispersed to the current habitat by moving large mammals (e.g., macaques and wild boars) through feces after consuming its delicious fruits.

Habitat filtration, niche differentiation, and interspecific competition are the three important processes that shape community species composition and community structure dynamics. According to Darwin (1859), plants with close relatives tend to colonize the same habitat because of their similar requirements. In the studied community, *C. oblonga* was closely related to *S. chinensis* and four other species of the Rosaceae family (Figure 6). These six closely related species were the most dominant in the studied community, accounting for 82.96% of the RIV of all shrubs (Table 2). Therefore, naturalization of *C. oblonga* supports the pre‐adaptation hypothesis, which states that alien species closely related to native ones are more likely to invade, naturalize, and dominate the local communities (Li, Cadotte, et al., 2015). Simultaneously, however, coexisting species experience strong interspecific competition because of their similar resource requirements (Castagneri et al., 2008). In subtropical steep rocky mountain habitats (Wen et al., 2015), shrublands are dominated by *S. chinensis*, which is the closest relative of *C. oblonga* (Figure 6). In the studied community, however, the *S. chinensis* population is declining and gradually being replaced by *C. oblonga* (Figures 3 and 4), supporting the conclusion that “the naturalization of alien species severely harms closely related native species, leading to their local extinction” (Li, Cadotte, et al., 2015). However, the link between the degree of phylogenetic relatedness and strength of competition remains controversial (Bezeng et al., 2015; Jones et al., 2013; Li, Cadotte, et al., 2015). Therefore, long‐term monitoring studies focusing on native community dynamics and alien plant populations may offer critical data to interpret the naturalization of *C. oblonga*.

Furthermore, the geographical climate of the Irano‐Turanian region—the center of origin of *C. oblonga*—is characterized by low precipitation and prolonged drought periods (Ghafari et al., 2018), similar to the environmental conditions at the top of the Baozhong Mountain, where solar radiation is intense, summers and autumn are dry, and habitats are barren and arid. Consequently, we speculated that climate similarity may be one of the major factors supporting the self‐sustaining population of these trees in the studied habitat. However, comparative analyses of soil, topography, and climate factors are warranted to draw a definitive conclusion in the future.

## 5 CONCLUSION

The present study reported on the naturalization of *C. oblonga* in China for the first time and systematically investigated its natural community structure and population dynamics. The identified community dominated by *C. oblonga* is at the intermediate or later stage of succession and may develop into a plagioclimax community in steep rocky habitats of the Baozhong Mountain. Religious exchange and climatic similarity with the original habitat may be the key drivers of the successful naturalization of this species in China. From our findings, alien species closely related to native ones are more likely to invade, naturalize, and dominate local habitats. In the present article, we describe comprehensive observations on the community and population dynamics as well as on the potential drivers of a naturalized ancient fruit tree in China; our work may serve as a reference for future research on the naturalization processes of invasive species at fine scales and, eventually, the applied management and utilization of these naturalized species.

## AUTHOR CONTRIBUTIONS

**Yong Xie:** Conceptualization (equal); data curation (equal); funding acquisition (equal); investigation (equal); project administration (equal); supervision (equal); writing – original draft (equal). **Jiaxiang Li:** Conceptualization (lead); data curation (lead); funding acquisition (equal); investigation (equal); methodology (lead); project administration (lead); supervision (equal); writing – original draft (equal). **Lijuan Zhao:** Conceptualization (equal); formal analysis (equal); funding acquisition (equal); writing – review and editing (equal). **Wenqian Liu:** Formal analysis (equal); investigation (equal); methodology (equal); software (equal). **Qunlong Gong:** Formal analysis (equal); investigation (equal); writing – review and editing (equal). **Mengda Deng:** Formal analysis (equal); investigation (equal); project administration (equal); software (equal). **Mohan Zhao:** Investigation (equal); software (equal). **Song Huang:** Formal analysis (equal); investigation (equal); writing – original draft (equal).

## Data Availability

The data that support the findings of this study are available from the corresponding author (Jiaxiang Li) upon reasonable request.
